# Optimized nitrogen management enhances lodging resistance by modulating stem physicochemical traits in oats

**DOI:** 10.1186/s12870-026-08840-z

**Published:** 2026-05-01

**Authors:** Ruifang Zhang, Rui Wu, Wenhui Liu, Guoling Liang, Zeliang Ju, Wen Li

**Affiliations:** 1 Key Laboratory of Excellent Forage Germplasm Resources Utilization in Qinghai-Tibet Plateau, College of Animal Husbandry and Veterinary Sciences, Qinghai University, Xining, 810016 Qinghai China; 2 Qinghai-Tibet Plateau Germplasm Resources Research and Utilization Laboratory, Xining, 810016 Qinghai China

**Keywords:** oats, Nitrogen management, Lodging, Carbon and nitrogen balance, SEM, TOPSIS

## Abstract

**Background:**

Oat (Avena sativa L.) is a critical dual-purpose crop for grain and forage, yet its yield potential is frequently constrained by stem lodging exacerbated by sub-optimal nitrogen (N) management. While the general physiological basis of lodging is known, the precise quantitative contributions of specific stem physicochemical traits under varying N regimes remain underexplored, particularly in unique high-altitude agroecosystems. To address this gap, we conducted a two-year field trial (2018–2019) in the rain-fed Qinghai-Tibet Plateau, evaluating two oat cultivars with contrasting lodging resistance (LENA, resistant; QY2, susceptible). Across six N rates (0, 60, 120, 180, 240, and 300 kg·ha⁻¹), we quantified structural components (lignin, cellulose), non-structural carbohydrates (soluble sugars, starch), and mineral elements (Ca, K, Si, Mg) in the second basal internode at the milk stage. Key determinants of stem strength were isolated using hierarchical partitioning, structural equation modeling (SEM), and TOPSIS analysis.

**Results:**

Lodging severity and stem physicochemical profiles were significantly driven by N rate, genotype, and their interaction. Increased N application elevated soluble protein levels but concurrently suppressed the accumulation of lignin, cellulose, and key minerals (Si, Ca, K). Hierarchical partitioning identified Ca, K, Si, soluble sugars, and lignin as the primary determinants, collectively explaining 87% of the variance in the lodging index. Furthermore, SEM revealed that genotype and high N inputs indirectly exacerbated lodging risk by downregulating the accumulation of these critical structural and mineral components. Total standardized effects on the lodging index were − 0.209 (genotype), 0.310 (N rate), and 0.156 (interaction).

**Conclusions:**

Based on the comprehensive production and lodging resistance performance, it is suggested that in the study area and similar ecological areas of the Qinghai-Tibet Plateau, the suitable nitrogen application rate of the lodging-resistant variety LENA should be 180 kg·hm^− 2^, while that of the lodging-prone variety QY2 should be controlled below 60 kg·hm^− 2^. These findings provide a physiological framework for rational nitrogen management and offer novel mechanistic insights into oat lodging resistance.

**Graphical Abstract:**

**Figure:**
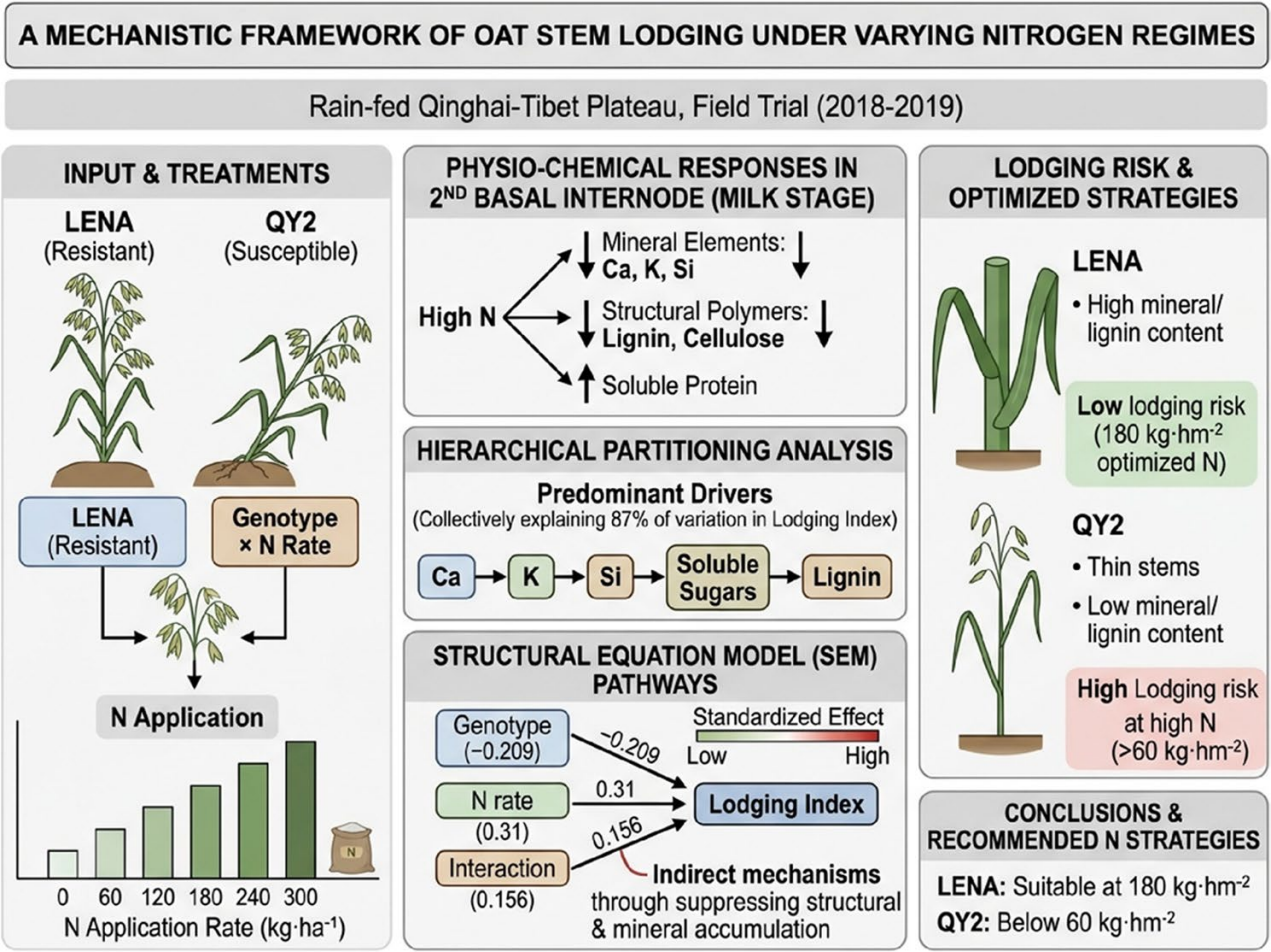
The proposed physiological and biochemical mechanisms of nitrogen-regulated lodging resistance in oats within the alpine rain-fed region of the Qinghai-Tibet Plateau.

## Introduction

Nitrogen (N) is an essential macronutrient that governs crop physiology and productivity. While optimal N application is critical for enhancing photosynthesis, biomass accumulation, and grain protein content [1, 2], it inherently presents a trade-off in crop performance. In intensive agricultural systems, the pervasive use of excessive N inputs not only exacerbates environmental footprints but also disrupts the allometric balance between vegetative growth and mechanical support, thereby predisposing crops to lodging [3]. Particularly in China, where oats serve as a crucial dual-purpose crop for food and forage, high-yielding cultivation paradigms heavily rely on intensive water and fertilizer inputs. Consequently, stem over-elongation and compromised structural integrity are prevalent, frequently triggering severe lodging events [4]. Such lodging severely constrains oat production by disrupting canopy architecture and impeding photosynthate translocation. This ultimately results in substantial yield penalties and quality deterioration, while simultaneously complicating mechanized harvesting [5]. Given that inappropriate N application rates remain a primary catalyst for lodging, a critical challenge in modern oat agronomy is to decouple yield maximization from lodging risk via precision N management strategies.

Stem lodging resistance is fundamentally dictated by biomechanical strength, which arises from the synergistic accumulation of cell wall structural components, mineral nutrients, and non-structural carbohydrates. Lignin and cellulose serve as the primary structural components of the cell wall, and their content directly determines stem flexural strength [6]. Mineral nutrients such as silicon (Si) and potassium (K) enhance stem rigidity by promoting epidermal silicification and regulating cellular osmotic potential [7]. Non-structural carbohydrates, such as starch and soluble sugars, not only increase stem mass density but also serve as key substrates for cell wall biosynthesis [8]. However, excessive nitrogen (N) application significantly disrupts these processes via the carbon-nitrogen (C-N) metabolic balance: high N input forces photosynthetic products to preferentially flow to nitrogen metabolic sinks (e.g., protein synthesis), resulting in a shortage of the carbon skeletons required for cell wall construction [9]. Concurrently, the rapid accumulation of biomass induced by high N levels produces a growth dilution effect, resulting in a significant decrease in the concentration of structural mineral elements per unit dry weight [10]. Although N-mediated regulation of lodging has been extensively studied in cereal crops such as rice (*Oryza sativa* L.) and wheat (*Triticum aestivum* L.) [3, 11, 12], systematic investigations in oat grown under the cold, rain-fed conditions of the Qinghai–Tibet Plateau remain scarce. Unlike staple cereals, oat is a dual-purpose crop for both grain and forage; thus, lodging not only disrupts grain filling but also compromises forage quality and mechanized harvesting efficiency. Lodging is a complex trait shaped by the coordinated action of morphological, physiological, and biomass-related factors. Furthermore, strong collinearity among physicochemical traits makes it difficult for conventional correlation analysis or linear regression to accurately quantify their independent effects or to elucidate the pathways by which N influences lodging through key traits. Consequently, there remains a lack of systematic quantification regarding the relative contributions of different physicochemical traits to lodging, the key physiological pathways involved in N-mediated lodging regulation, and the optimal N thresholds required for different genotypes to balance stem material accumulation with lodging resistance under specific ecological conditions. To address these gaps, this study utilized two oat cultivars with contrasting lodging resistance grown on the rain-fed Qinghai–Tibet Plateau. We aimed to establish an integrated framework linking N application, stem physicochemical traits, lodging risk, and N optimization, thereby providing a theoretical basis for coordinating efficient stem material accumulation and lodging resistance in regional oat production.

## Result

### Variations in stem characteristics between oat cultivars with contrasting lodging resistance

**Fig. 1:**
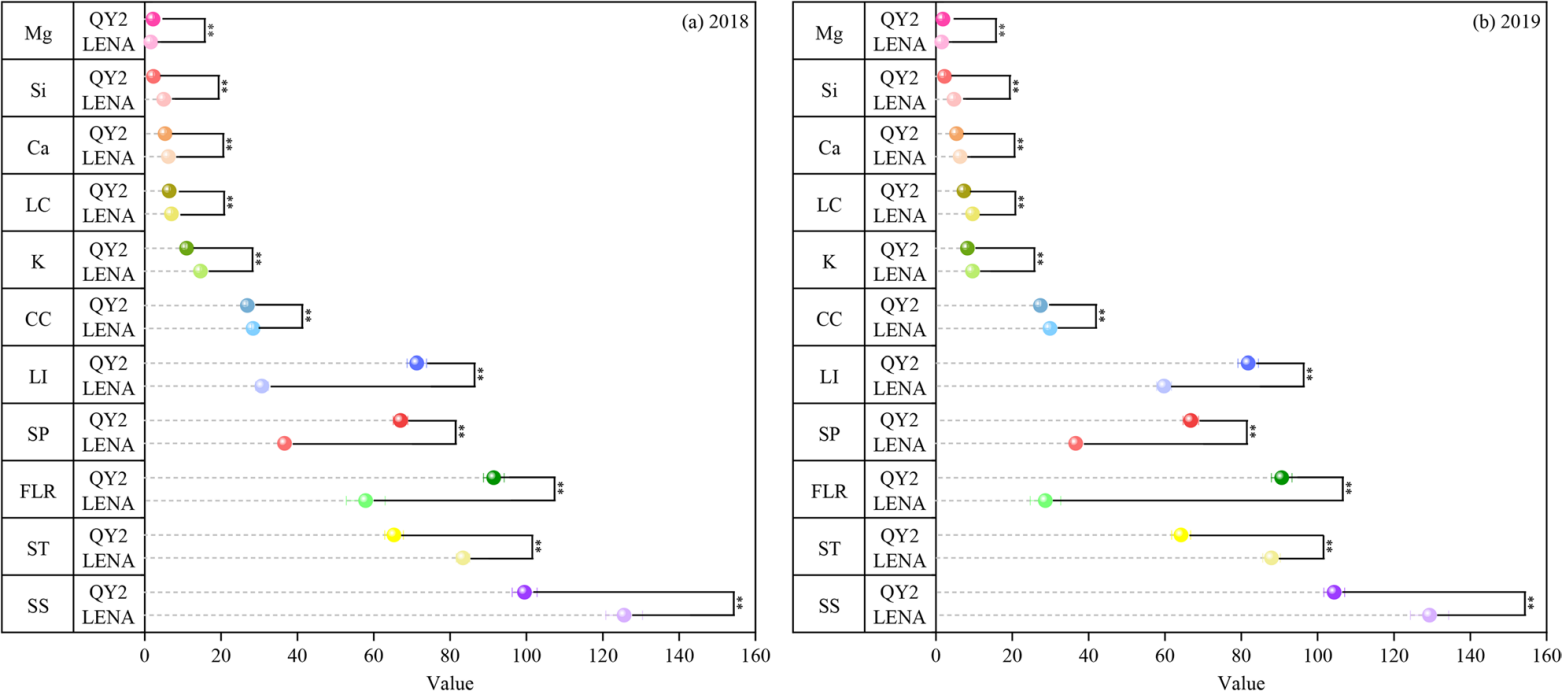
Differences in stem physicochemical properties, lodging index, and field lodging rate between two oat varieties (LENA and QY2) in (**a**) 2018 and (**b**) 2019. Magnesium (Mg); Silicon (Si); Calcium (Ca); Lignin Content (LC); Potassium (K); Cellulose Content (CC); Lodging Index (LI); Soluble Protein (SP); Field Lodging Rate (FLR); Starch (ST), Soluble Sugar (SS). ** indicates that there is a significant difference at the *P* < 0.01

Experimental data from the two-year study (2018–2019) revealed highly significant differences (*P* < 0.01) across all measured traits between the lodging-resistant cultivar LENA and the lodging-susceptible cultivar QY2 (Fig. 1). Compared with LENA, the susceptible cultivar QY2 exhibited markedly lower levels of structural components (LC, CC) and non-structural carbohydrates (SS, ST). Conversely, LENA maintained a significantly higher accumulation of key mineral elements (Si, Ca, and K) known to enhance mechanical strength. In contrast, the magnesium (Mg) and soluble protein contents in the stems of QY2 were significantly higher than those in LENA.

### Impact of N fertilization on oat lodging incidence and index

**Fig. 2:**
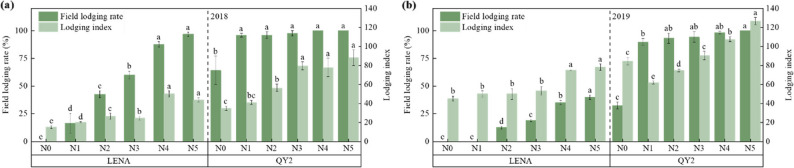
Response of field lodging rate and lodging index to varying nitrogen dosages in two oat cultivars. **a** indicates 2018 data; **b** represents 2019 data. The dark green column represents the field lodging rate (left axis), and the light green column represents the lodging index (right axis). N0-N5 represents different amounts of nitrogen fertilizer. Different lowercase letters in the column indicate significant differences between treatments at the *P* < 0.05, the same below

Nitrogen (N) application significantly affected the lodging phenotypes of the oat plants. Generally, as the N application rate increased from N0 to N5, the field lodging rate and lodging index of both cultivars exhibited a significant upward trend in 2018 (Fig. 2a) and 2019 (Fig. 2b), peaking under the high-N treatments (N4–N5). However, the cultivars displayed distinct response patterns to N fertilization. The lodging-susceptible cultivar QY2 was highly sensitive to N input. In 2018, its field lodging rate reached 63.27% even without N application (N0). Under the N1 treatment, this rate surged to 95.97%, and the corresponding lodging index was approximately 1.74 times that of LENA at the same N level (35.03 vs. 20.13). In contrast, the lodging-resistant cultivar LENA demonstrated high N tolerance; no obvious lodging occurred under the N0 treatment in either growing season or under the N1 treatment in 2019. Even under the maximum N application (N5) in 2019, the field lodging rate of LENA remained significantly lower than that of QY2. Interestingly, although the field lodging rate generally increased alongside the lodging index, LENA’s lodging index reached 50.19 under the N1 treatment in 2019, yet its actual field lodging rate remained at zero.

### Effect of N application on the physicochemical traits of oat stems

**Fig. 3:**
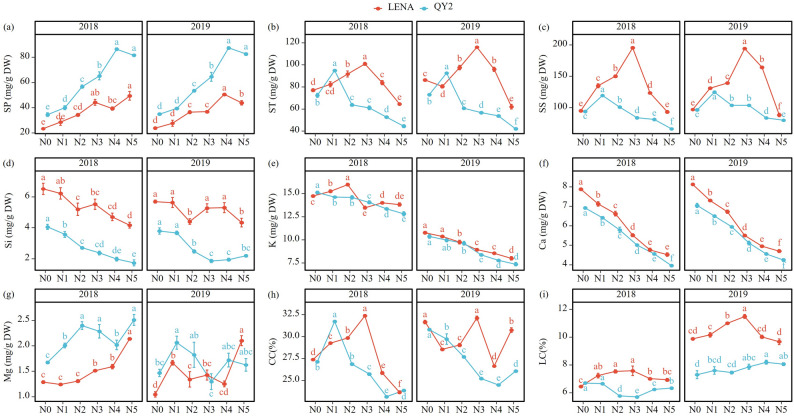
Effects of different nitrogen levels on stem physicochemical properties and mineral elements of two oat varieties (LENA and QY2) in 2018 and 2019. Changes in (**a**) SP (Soluble Protein, mg/g DW), (**b**) ST (Starch, mg/g DW), (**c**) SS (Soluble Sugar, mg/g DW), (**d**) Si (Silicon, mg/g DW), (**e**) K (Potassium, mg/g DW), (**f**) Ca (Calcium, mg/g DW), (**g**) Mg (Magnesium, mg/g DW), (**h**) CC (Cellulose Content, %), and (**i**) LC (Lignin Content, %) under six nitrogen treatments (N0 to N5) are presented. The red line represents the LENA variety, and the blue line represents the QY2 variety. DW indicates dry weight. Different lowercase letters indicate statistically significant differences among the different nitrogen treatments within the same oat variety and year (*P* < 0.05)

Soluble protein (SP) content increased in tandem with the N application rate (Fig. 3a). In 2018, as the N rate increased from N0 to N5, the SP content in QY2 rose from 34.17 mg/g to 81.58 mg/g, representing a 138.7% increase. LENA exhibited a similar but more moderate upward trend; its SP content increased from 23.33 mg/g at N0 to 49.29 mg/g at N5, reflecting a 111.3% increase. Similar patterns were observed in 2019.

Starch (ST) and soluble sugar (SS) contents initially increased but subsequently declined with escalating N application rates (Fig. 3b, c). The two cultivars differed significantly in SS accumulation (*P* < 0.01) (Table 1). QY2 was highly sensitive to N input, with its SS reaching a maximum under the N1 treatment—approximately 2.2 times higher than that under the N5 treatment. Conversely, SS in LENA peaked under the N3 regime, showing a significant increase compared to all other treatments. Specifically, in 2018 and 2019, the peak SS values for LENA were 56.27% and 86.97% higher, respectively, than those under the N5 treatment, which yielded the lowest content. The variation in ST content mirrored that of SS: ST in QY2 and LENA peaked under the N1 and N3 treatments, respectively, significantly outperforming other N levels. Demonstrating higher sensitivity to N input, the SS content of QY2 dropped precipitously from 119.34 mg/g under the N1 treatment to 66.21 mg/g under the N5 treatment in 2018. In contrast, LENA exhibited greater N tolerance; in 2019, its SS content under the N5 treatment (88.18 mg/g) remained significantly higher than that of QY2 (79.93 mg/g).

**Table 1:** Analysis of variance

Characteristics	Variety	Nitrogen Fertilizer	Variety×Nitrogen Fertilizer
SP	1131.3**	305.91**	51.85**
ST	1503.01**	288.3**	239.34**
SS	4022.31**	900.93**	620.78**
Si	748.77**	43.52**	4.47**
K	54.36**	158.25**	5.68**
Ca	413.17**	1032.21**	11.98**
Mg	101.56**	21.58**	7.75**
CC	792.6**	453.31**	247.7**
LC	861.74**	6.95**	26.15**
FLR	2785.36**	306.11**	59.87**
LI	535.27**	181.41**	20.43**

The values indicate the F-statistics. *, **Denote significant differences at *P* < 0.05 and *P* < 0.01, respectively

The N application rate significantly impacted stem mineral concentrations. Over the two-year period, the silicon (Si) content of both cultivars exhibited a downward trend as N input increased, though the decline was more pronounced in QY2 than in LENA (Fig. 3d). In 2018, the Si content in QY2 decreased significantly from 4.05 mg/g (N0) to 1.72 mg/g (N5). Meanwhile, LENA maintained relatively high Si levels across all treatments; although its content decreased from 6.52 mg/g (N0) to 4.16 mg/g (N5), this N5 value remained significantly higher than that of QY2. Following a pattern similar to Si, potassium (K) and calcium (Ca) contents also declined with increasing N application (Fig. 3e, f). In 2018, the K and Ca contents in QY2 under the N5 treatment were 17.88% and 74.94% lower than those under the N0 treatment, respectively. For LENA, K content decreased by only 6.73%, while the reduction in Ca content was comparable to that of QY2. Conversely, N fertilization exerted a significant positive influence on magnesium (Mg) accumulation (*P* < 0.01), with levels generally rising in parallel with the N gradient (Fig. 3g).

Furthermore, N application significantly affected lignin (LC) and cellulose (CC) contents (*P* < 0.01) (Table 1). As N levels increased, both CC and LC contents trended downward in both cultivars (Fig. 3h, i). During the 2018 season, CC levels in LENA and QY2 under the N5 regime were reduced by 15.78% and 13.73%, respectively, relative to the N0 control. Significant genotypic differences were also observed in LC accumulation; for instance, under the N5 regime in 2019, the LC content in LENA was 9.66%, markedly surpassing the 8.05% recorded for QY2.

### Responses of stem physicochemical traits and lodging to N dosage and cultivar differences

Table 1 details the main and interactive effects of variety (V) and N application rate (N) on the mineral nutrients, structural components, non-structural components, and lodging incidence of oat stems. Variety exerted a highly significant effect (*P* < 0.01) on all measured parameters, with the most pronounced impact observed on soluble sugar (SS) content (*F* = 4022.31, *P* < 0.001), followed by the field lodging rate (*F* = 2785.36, *P* < 0.001). Similarly, the N application rate demonstrated a highly significant main effect across all indicators (*P* < 0.01). Its strongest influence was on calcium (Ca) content (*F* = 1032.21, *P* < 0.001), followed closely by SS accumulation (*F* = 900.93, *P* < 0.001). Furthermore, a highly significant variety-by-nitrogen (V × N) interaction was detected for both lodging characteristics (field lodging rate and lodging index) and stem physicochemical traits (*P* < 0.01). Among these interactive effects, the greatest variance was again driven by SS content (*F* = 620.78, *P* < 0.001).

### Screening of key physicochemical factors affecting the lodging index

**Fig. 4:**
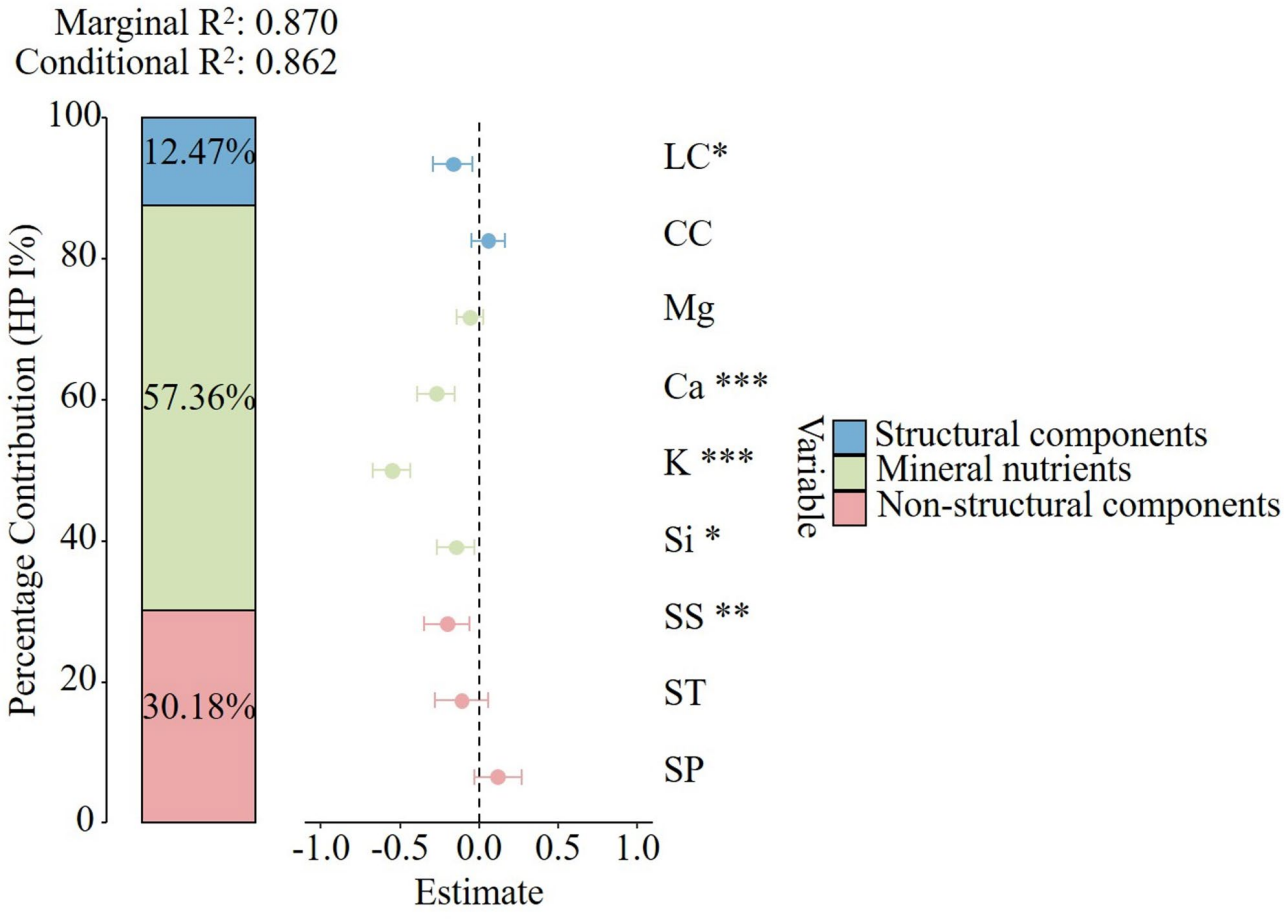
Relative contributions and effects of stem physicochemical properties and mineral nutrients on the Lodging index (LI) based on hierarchical partitioning and mixed-effects models. The stacked bar chart on the left illustrates the percentage contribution (HP I%) of three parameter categories (structural components, mineral nutrients, and non-structural components). The forest plot on the right displays the mixed-effects model estimates (dots) and their corresponding 95% confidence intervals (error bars) for each individual predictor. Evaluated predictors include structural components: LC (Lignin content) and CC (Cellulose content); mineral nutrients: Mg (Magnesium), Ca (Calcium), K (Potassium), and Si (Silicon); and non-structural components: SS (Soluble sugar), ST (Starch), and SP (Soluble protein). Marginal R² and conditional R² represent the variance explained by the fixed effects and by the combination of fixed and random effects, respectively. Asterisks denote statistical significance, *: *P* < 0.05, **: *P* < 0.01, ***: *P* < 0.001

To address the issue of multicollinearity among the physiological and biochemical indicators, we performed a hierarchical partitioning analysis based on a linear mixed-effects model (Fig. 4). The model exhibited high explanatory power for the lodging index (marginal *R*² = 0.870), indicating that the selected traits accounted for the vast majority of the phenotypic variation, with a negligible influence from random effects. The analysis revealed distinct differences in the independent contributions of the three trait categories, ranking as follows: mineral nutrients > non-structural components > structural components. Specifically, mineral nutrients (Si, K, Ca, and Mg) were the dominant drivers, with an independent contribution of 57.36%, which was substantially higher than that of the other two categories. Non-structural carbohydrates (SS, ST, and SP) followed, explaining 30.18% of the variation, whereas structural components (LC and CC) contributed the least (12.47%). At the individual trait level, Ca, K, and SS exerted highly significant effects on the lodging index (*P* < 0.01), while Si and LC were significant at the *P* < 0.05 level. Consequently, these five variables were identified as the key regulators of oat lodging.

### Physiological pathway analysis of N-regulated oat lodging

**Fig. 5:**
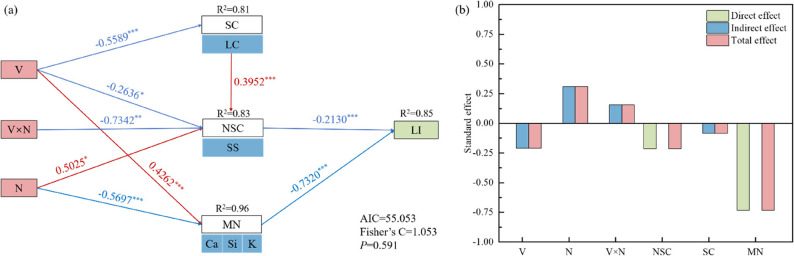
Structural equation model and path analysis of the effects of nitrogen fertilizer and variety on the lodging index. **a** Structural equation model (SEM) showing the direct and indirect effects of Variety (V), nitrogen application rate (N), and their interaction (V × N) on LI through Structural components (SC), non-structural components (NSC), and mineral nutrients (MN). Blue and red arrows indicate significant negative and positive effects, respectively. The number next to the arrow is the standardized path coefficient. The value (R^2^) above the box represents the explanatory rate of the model to the variance of the variables. Significant level: * *P* < 0.05, ** *P* < 0.01, *** *P* < 0.001. **b** Represents the standardized direct, indirect, and total effects of each factor on LI

Building on the significant factors (LC, Ca, K, Si, and SS) identified via the hierarchical partitioning analysis, a piecewise structural equation model (SEM) was constructed to delineate the specific pathways through which variety (V), N application rate (N), and their interaction (V × N) regulate the lodging index via physicochemical traits (Fig. 5a). Goodness-of-fit metrics indicated that the model aligned exceptionally well with the data (Fisher’s *C* = 1.053, *P* = 0.591 > 0.05; Akaike Information Criterion [AIC] = 55.053), thereby validating the pathway analysis. The SEM successfully explained 85% of the total phenotypic variance in the lodging index (*R*² = 0.85).

Mineral nutrients (MN) and non-structural components (NSC) exerted robust, direct negative regulation on the lodging index, evidenced by path coefficients of -0.732 and − 0.213, respectively (*P* < 0.001). Lignin (SC) was predominantly modulated by soluble sugars (NSC), thereby exerting a significant indirect influence on lodging (coefficient: -0.0842; *P* < 0.001). Furthermore, the impact of variety, nitrogen dosage, and their interplay on oat lodging was mediated via alterations in lignin, soluble sugars, and mineral profiles (Ca, K, and Si), with total effect values of -0.209, 0.31, and 0.156, respectively (Fig. 5b).

### Comprehensive evaluation

**Fig. 6:**
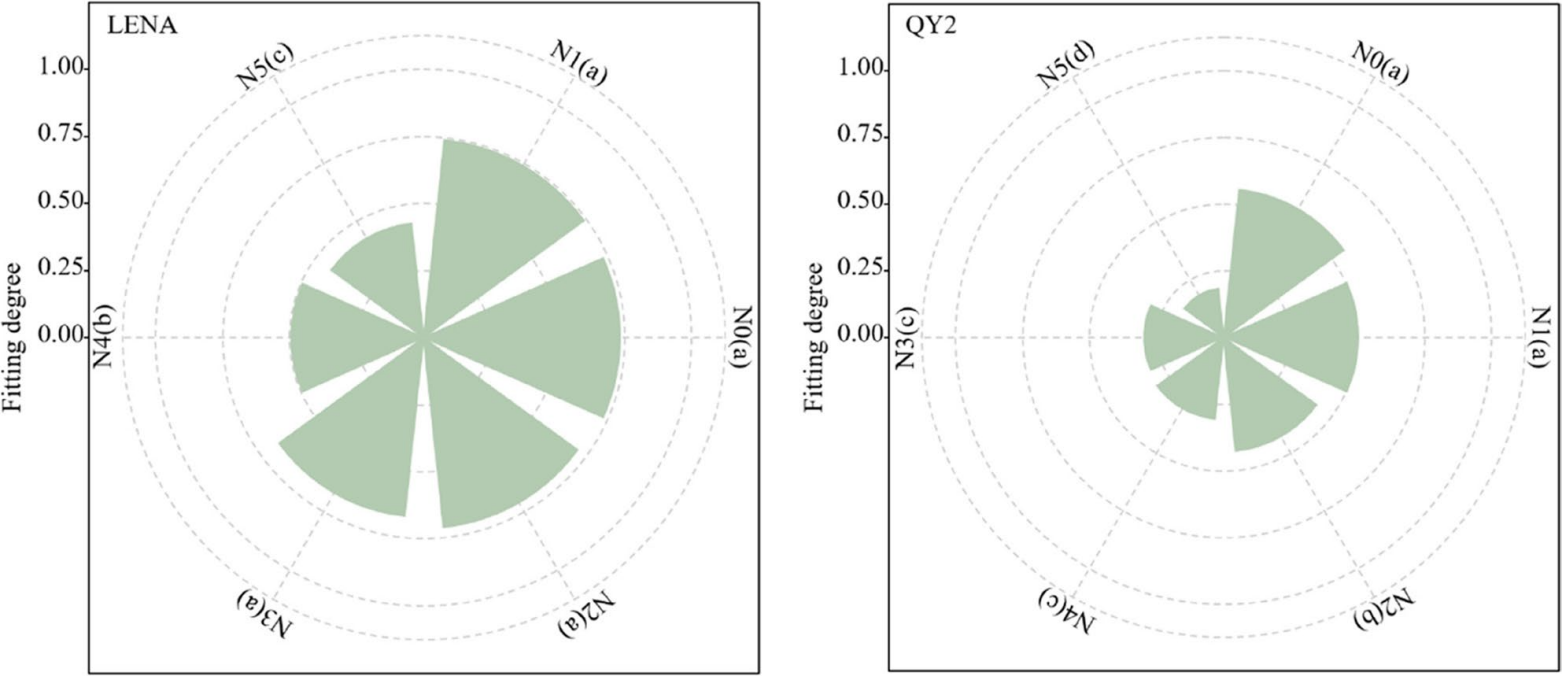
Comprehensive evaluation of important physicochemical indexes and Lodging Index of LENA (left) and QY2(right). Significant differences among treatments are denoted by distinct lowercase letters (*P* < 0.05)

Through a comprehensive evaluation of the lodging index and other key factors, our results indicated that the optimal N dosage was highly variety-dependent (Fig. 6). For LENA, the highest fitness score (0.740) was observed under the N1 treatment, although this was not significantly different from the scores obtained under the N0, N2, and N3 regimes. Conversely, the N5 treatment yielded the lowest score for LENA (0.427). In contrast, QY2 achieved its maximum fitness score under the N0 control (0.556), which showed no significant difference from that of the N1 treatment (0.501). Similar to LENA, the lowest score for QY2 was recorded under the N5 treatment (0.390).

## Discussion

### Nitrogen-driven ‘carbon–nitrogen trade-off’ and variety-specific responses

In this study, the distinct stem traits observed among oat varieties confirm that, in addition to agronomic practices, genetic background is a critical intrinsic determinant of lodging resistance [13]. Previous studies have demonstrated that lodging-resistant germplasm is typically characterized by elevated levels of Ca, K, Mg, Si, lignin, and hemicellulose [14]. Our results were largely consistent with these findings in terms of the accumulation of Ca, K, Si, crude fiber, and lignin in stems. However, a notable divergence was observed for Mg. Unexpectedly, the lodging-susceptible variety QY2 exhibited a significantly higher Mg content than the lodging-resistant variety LENA. This anomaly may be attributed to differences in Mg utilization efficiency. It has been reported that lodging-susceptible varieties often exhibit lower Mg use efficiency; under high N conditions, they may preferentially allocate resources toward N assimilation and K uptake, thereby suppressing Mg-related physiological functions (e.g., photosynthetic efficiency), ultimately resulting in its passive accumulation rather than effective metabolic utilization within plant tissues [15].

Notably, the lodging-resistant variety LENA accumulated significantly greater reserves of non-structural components (specifically starch and soluble sugars), markedly exceeding those of the susceptible variety QY2. Although this pattern contrasts with findings from studies conducted under no-N conditions [7, 16], when considered alongside the significantly higher soluble protein content observed in QY2, it likely reflects differential carbon–nitrogen metabolic responses induced by exogenous N input. According to the “Carbon-Nitrogen Balance Hypothesis,” high N availability promotes the preferential allocation of photosynthates toward nitrogen sinks (e.g., protein synthesis), thereby competitively constraining the incorporation of carbon skeletons into structural components (e.g., lignin and cellulose) [17–19]. In the present study, the susceptible variety QY2 exhibited pronounced N sensitivity, with N application markedly stimulating soluble protein synthesis (N pool) while depleting non-structural components (C pool). This intensified source–sink competition resulted in insufficient carbon availability for structural reinforcement during rapid stem elongation. Moreover, the “growth dilution effect” associated with accelerated biomass accumulation [10] further reduced the concentration of mechanically supportive elements (Ca, Si, and K) and lignin per unit dry weight. In contrast, the resistant variety LENA demonstrated superior metabolic homeostasis. N uptake in LENA appears to enhance photosynthetic source capacity rather than merely promoting vegetative expansion. Consequently, LENA maintained a sufficient pool of non-structural components and sustained the accumulation of structural materials even under high N conditions.

### Mechanical safety thresholds and the carbon–nitrogen balance in stem lodging

There is no simple linear correlation between the lodging index (LI) and the field lodging rate (FLR); rather, a “mechanical safety threshold” exists [20, 21]. In this study, although the FLR generally paralleled the LI, the lodging-resistant variety LENA exhibited an LI of 50.19 under the N1 treatment in 2019, yet its actual FLR remained at zero. This suggests that as long as the comprehensive mechanical strength of the stem—derived from structural components (e.g., lignin and cellulose) and silicification—is sufficient to withstand external bending moments, the plant remains within a “mechanically safe zone” despite an elevated risk index [22, 23]. However, this safety margin is dynamic and environment-dependent. Fundamentally, lodging results from the interplay between intrinsic factors (stem mechanical weakening) and extrinsic factors (environmental stress). In 2019, the absence of extreme weather events meant that the potential risk indicated by a high LI did not translate into actual lodging. In contrast, heavy rainfall during the early grain-filling stage in 2018 (30.4 mm on July 23; data from era5: https://cds.climate.copernicus.eu) acted as a critical trigger. The increased panicle weight resulting from water absorption shifted the center of gravity upward; this, compounded by the kinetic impact of the rain, forced plants already in a critical state to exceed their mechanical safety threshold. Further analysis revealed that the magnitude of this safety threshold reflects varietal differences in adaptability to N-induced carbon–nitrogen (C–N) imbalances. The lodging-susceptible variety QY2 is highly sensitive to N; the accumulation of its non-structural components peaked prematurely under low N conditions (N1) and was rapidly depleted to fuel vigorous N metabolism, leaving insufficient carbon skeletons for cell wall construction. Consequently, lodging occurred as the physical support fell below the safety limit. In contrast, the lodging-resistant variety LENA exhibited superior C–N homeostasis. Its accumulation peak for non-structural components shifted to the medium N level (N3), enabling the maintenance of higher Si and lignin contents even under severe N stress. This physiological advantage confers a higher safety threshold and greater buffering capacity, allowing LENA to maintain upright growth despite N-induced declines in mechanical properties.

### Correlations of the lodging index with variety, nitrogen dosage, and culm physicochemical traits

#### Mineral nutrients are the primary determinants of stem lodging resistance

To mitigate multicollinearity among physicochemical traits, hierarchical partitioning was employed to isolate inter-variable interference and quantify their independent contributions to the lodging index. Mineral nutrients (Si, K, and Ca) accounted for 57.36% of the variation in the lodging index, emerging as the primary determinants of stem strength. Previous reports have elucidated the mechanisms underlying these effects. Silicon, acting as a skeletal material within the cell wall, significantly enhances stem mechanical rigidity and toughness upon deposition, thereby reducing the lodging risk [24]. Calcium, a key component of pectin in the middle lamella, participates in cell wall assembly, enhancing intercellular adhesion and tissue compactness [25]. Potassium is critical for maintaining cell turgor, which promotes the development of thick-walled mechanical tissues [26]. Our results confirm that elevated stem concentrations of these elements enhance lodging resistance, thereby negatively regulating the lodging index.

#### Synergistic regulation of non-structural and structural components

Previous studies have often suggested that structural components such as lignin are the primary determinants of stem strength [27–29]. However, our findings diverge from this paradigm. Hierarchical partitioning revealed that the independent contribution of non-structural components (soluble sugars, starch, and soluble protein) to the lodging index (30.18%) markedly exceeded that of structural components (lignin and cellulose) (12.47%). Structural equation modeling (SEM) further elucidated this underlying mechanism: soluble sugars exerted a significant direct negative effect on the lodging index (path coefficient = − 0.213). While lignin also inhibited lodging, its influence was more complex, acting primarily indirectly and mediated by soluble sugars (path coefficient = − 0.0842). These results suggest that soluble carbohydrates not only regulate cellular osmotic potential to maintain stem turgor but also serve as a core substrate pool, indirectly modulating the synthesis of structural components (e.g., lignin) via metabolic flux partitioning [30, 31]. Consequently, evaluations of oat lodging resistance should prioritize not only lignin content but also the accumulation of non-structural components, particularly soluble sugars.

#### Physiological and biochemical pathways mediating the effects of N application and variety on lodging resistance

This study delineated a comprehensive pathway linking agronomic practices to physiological traits, and ultimately to lodging resistance. Varietal characteristics, N application rates, and their interactions acted as exogenous drivers, determining the lodging index by regulating soluble sugar accumulation, lignin biosynthesis, and mineral element (Ca, K, Si) uptake in the stem. Based on the total effect values from the SEM, the magnitude of lodging inhibition was: mineral nutrients (–0.732) > non-structural components (–0.213) > structural components (–0.0842). Rational N management optimizes the partitioning of photosynthates, driving the efficient translocation of non-structural components (e.g., soluble sugars)—which serve as fundamental carbon skeletons—to the secondary cell wall, thereby providing sufficient substrates for the biosynthesis of structural components like lignin. Conversely, excessive N application exacerbates source–sink competition, leading to a C–N metabolic imbalance. This drives the overconsumption of photosynthates for vegetative growth, thereby competitively inhibiting mechanical tissue construction [32]. Furthermore, alongside confirming the synergistic regulation of soluble sugars and lignin, this study highlighted the decisive role of mineral elements. Effective K accumulation in the stem helps maintain high cell turgor pressure [33], which is fundamentally required for upright rigidity. Under optimal N levels, stem K ensures vertical stiffness via this turgor maintenance. Simultaneously, the substantial accumulation of Si and Ca integrates extensively with cell wall structural components (lignin and cellulose) [34, 35], directly enhancing the physical support of the stem.

The contrasting performance of lodging-susceptible and lodging-resistant varieties across varying N levels essentially stems from their differential sensitivities to these metabolic pathways [11]. Therefore, the physiological basis of utilizing rational N application to mitigate lodging risk lies in optimizing stem enrichment of Si, K, and Ca, alongside the rational partitioning of soluble sugars and lignin, to construct a high-strength physical structure. This conclusion aligns with Mulder et al.’s classical view [36] regarding the synergy between mineral nutrients and carbon metabolites, providing a deeper, mechanistic understanding of N-mediated lodging resistance in oats.

#### Expanded significance of this study

While the general paradigm of increased lodging risk under N fertilization is well established in crops like rice and wheat, the significance of the present study lies in extending this framework to the rain-fed oat production systems of the Qinghai–Tibet Plateau. We further refined this understanding by quantifying multi-trait contributions, elucidating metabolic pathways, and proposing region-specific N management strategies. Compared to previous research, this study not only identified Ca, K, Si, soluble sugars, and lignin as core determinants of oat lodging but also elucidated significant variety-specific responses to N input. This facilitates the development of variety-tailored N management recommendations for the Qinghai–Tibet Plateau and similar agroecological zones. Ultimately, this suggests that the core of N management in this ecological zone is not merely lodging reduction, but rather the stabilization of stem structure to maximize the specific yield potential of distinct oat varieties.

### Optimal nitrogen application rates for distinct oat varieties

Rational N management is crucial for synergistically promoting oat growth, improving forage quality, and enhancing lodging resistance [37, 38]. Previous studies have shown that appropriate N application rates can optimize population structure, promote the rational distribution of structural and non-structural stem components, enhance mechanical strength, and consequently mitigate lodging risk [39]. Our results demonstrated significant varietal differences in N response. The lodging-susceptible variety QY2 proved highly sensitive to N fertilizer; its comprehensive evaluation scores under N0 and N1 were significantly higher than those under high N treatments, with no statistical difference between the N0 and N1 levels. In contrast, the lodging-resistant variety LENA exhibited greater N tolerance. Although its evaluation score peaked under the N1 treatment (0.74), this did not differ statistically from the N0, N2, or N3 treatments. While low N treatments can help maintain certain traits that enhance lodging resistance, N availability remains the primary driver of biomass accumulation. Suboptimal N application restricts normal plant development and material accumulation, thereby severely limiting crop dry matter yield and basal biomass [40]. Therefore, provided that lodging risk is not significantly elevated, prioritizing higher N application rates is essential to meet physiological metabolic demands, ensure stem material enrichment, and fully realize the crop’s growth potential.

In summary, oat production should adhere to variety-specific fertilization principles, seeking the optimal balance between efficient stem material accumulation and lodging resistance. For the lodging-susceptible variety QY2, N1 represents a rational N application threshold for maintaining basic physiological metabolism alongside lodging resistance. Conversely, for the lodging-resistant variety LENA, N3 is the ideal N level, maximizing mineral nutrient uptake and the enrichment of C and N metabolites while still ensuring structural stability. Future research should further explore optimal N application rates tailored to specific oat varieties and distinct environmental conditions to achieve the ultimate balance among physiological metabolism, forage quality, and lodging resistance. Ultimately, the optimal N application rates proposed in this study are not solely predicated on minimizing lodging risk; rather, they are based on the differential varietal responses to N under the ecological conditions of the Qinghai–Tibet Plateau, comprehensively accounting for stem physicochemical accumulation, lodging resistance stability, and overall yield potential.

## Conclusion

Nitrogen fertilizer significantly influenced the physicochemical traits and lodging dynamics of oats (*P* < 0.05). Optimizing N management enhances lodging resistance by modulating the accumulation of mineral nutrients, soluble sugars, and lignin. Specifically, the stem concentrations of Ca, K, Si, soluble sugars, and lignin were strongly correlated with lodging resistance, indicating their utility as reliable evaluation indicators. Comprehensive analysis revealed that for the lodging-resistant variety LENA and the lodging-susceptible variety QY2, the optimal balance between efficient stem material accumulation and lodging resistance was achieved under the N3 (180 kg·hm⁻²) and N1 (60 kg·hm⁻²) treatments, respectively. These represent the ideal N application rates for oat production in the study area and are recommended for broader application across similar agroecological zones of the Qinghai–Tibet Plateau.

## Materials and methods

### Study area

**Fig. 7:**
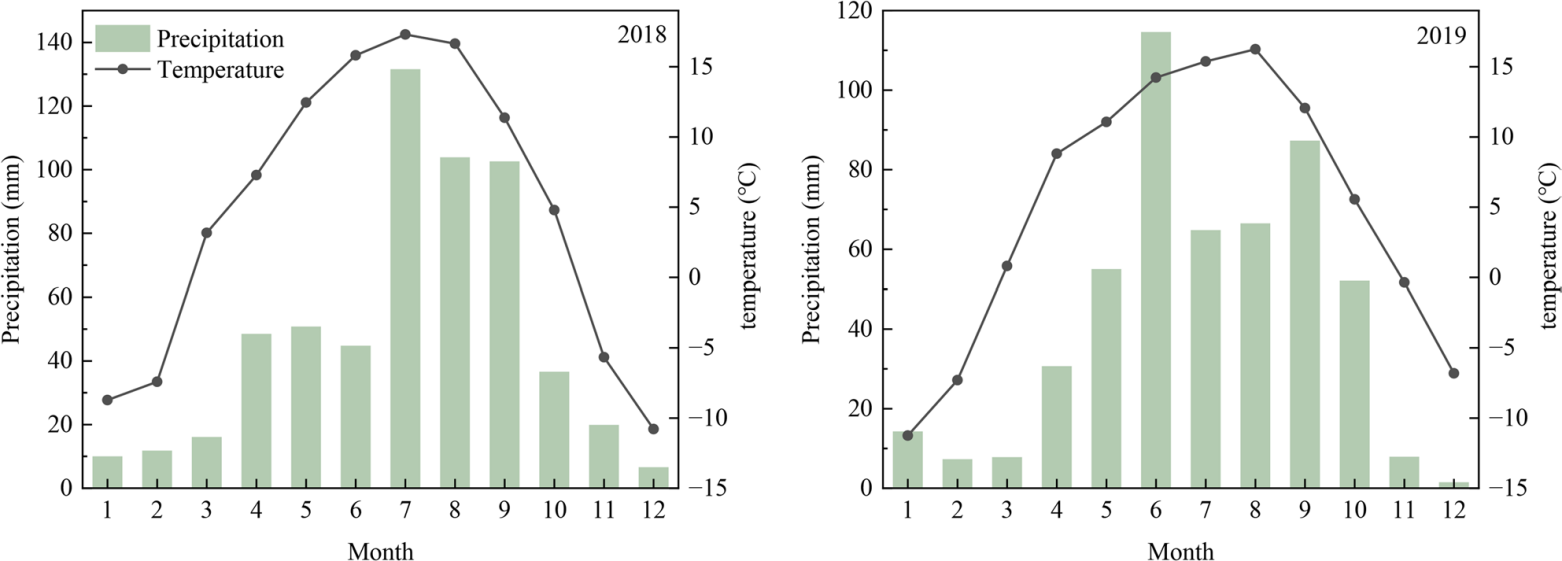
Precipitation and temperature in 2018 and 2019

Field experiments were conducted in Ganhetan Town (Huangzhong District, Xining City), situated in the hinterland of the Qinghai–Tibet Plateau (36°30′57″N, 101°33′20″E; elevation 2628 m). This region features a typical plateau continental climate characterized by a short frost-free season (90–120 days) and intense solar radiation (2550–2600 annual sunshine hours). The area is cool and semi-arid, with a mean annual temperature of 3.5–4.5 °C and significant diurnal fluctuations (12–16 °C). Precipitation averages 450–500 mm annually—primarily concentrated between June and August—while evaporation rates are 3 to 4 times higher. The dominant soil type is chestnut soil, and its specific physicochemical properties are detailed in Table 2. Meteorological conditions during the 2018–2019 growing seasons (sourced from ERA5, https://cds.climate.copernicus.eu) are illustrated in Fig. 7.

**Table 2:** Physicochemical properties of the soil at the experimental site prior to sowing

Index	Organic matter (g·kg⁻¹)		Total nitrogen (g·kg⁻¹)	Alkaline hydrolysis nitrogen (mg·kg⁻¹)	Available phosphorus (mg·kg⁻¹)	Rapidly available potassium (mg·kg⁻¹)	pH
value		58.24	3.12	54.28	4.83	112.45	7.8–8.3

### Test materials

The oat varieties evaluated in this study were LENA (*Avena sativa* L. cv. LENA) and Qingyin No. 2 (*Avena sativa* L. cv. QY2), provided by the Qinghai Academy of Animal Husbandry and Veterinary Sciences. These specific varieties were carefully selected based on multi-year preliminary field observations on the Qinghai–Tibet Plateau, which demonstrated stable and highly significant contrasts in their lodging-related traits. Specifically, LENA is a typical lodging-resistant variety characterized by superior stem mechanical strength, whereas QY2 is notably lodging-susceptible. Both are widely cultivated, premium dual-purpose oats exhibiting excellent ecological adaptability in this alpine region. Utilizing these two contrasting and regionally representative varieties provides an ideal comparative model to elucidate the physiological mechanisms underlying lodging, ensuring that the proposed N management strategies are practically applicable to local agricultural production. The morphological and agronomic characteristics of the materials are detailed in Table 3. The N source utilized was urea (46% N).

**Table 3:** Characteristics of the tested oat varieties

Variety	Characteristics
LENA	Plant height 90–110 cm; medium-late maturing; open panicle; yellow, spindle-shaped grains; 2–5 tillers per plant; lodging-resistant.
QY2	Plant height 120–140 cm; early maturing; open panicle; yellow, lanceolate grains; 2–3 tillers per plant; lodging-susceptible.

### Experimental design

A field experiment was conducted during the 2018–2019 growing seasons using a randomized complete block design (RCBD). Nitrogen was applied at six distinct rates: 0, 60, 120, 180, 240, and 300 kg N hm⁻² (designated as N0–N5, respectively). The experiment comprised 12 treatment combinations with three biological replicates, totaling 36 plots. Each plot covered an area of 15 m² (3 m × 5 m). All N treatments were broadcast as a basal application prior to sowing. The seeding rate was adjusted based on the 1000-grain weight to achieve a target stand density of 4.5 × 10⁶ seedlings hm⁻². Plots were established on a field previously cropped with highland barley, utilizing a 20 cm row spacing and 1 m alleyways between blocks. A uniform basal fertility level was established using 150 kg·hm⁻² of diammonium phosphate prior to seeding. Hand-weeding was strictly enforced during the seedling, tillering, and jointing stages to eliminate biotic interference.

### Indicators and methods of determination

#### Main agronomic traits

At the milk stage, to eliminate potential edge effects, six plants exhibiting uniform growth were randomly sampled strictly from the inner central area of each plot. This yielded a total of 18 plants per N treatment across the three replicates, which were subsequently used to evaluate the following parameters:

Height of the center of gravity (HCG): The distance from the base of the stem to the equilibrium fulcrum when the entire main stem (including leaves, sheaths, and spikes) was balanced horizontally.

Aboveground fresh weight (AFW): The total fresh weight of the plant’s aboveground components (stems, leaves, sheaths, and spikes).

#### Field Lodging Eate (FLR)

During the milk stage, the lodged area within each N treatment plot was visually assessed to calculate the FLR [41], using the following formula:

$$\:FLR\left(\%\right)=\frac{\mathrm{A}\mathrm{r}\mathrm{e}\mathrm{a}\:\mathrm{o}\mathrm{f}\:\mathrm{l}\mathrm{o}\mathrm{d}\mathrm{g}\mathrm{i}\mathrm{n}\mathrm{g}}{\mathrm{A}\mathrm{r}\mathrm{e}\mathrm{a}\:\mathrm{o}\mathrm{f}\:\mathrm{s}\mathrm{m}\mathrm{a}\mathrm{l}\mathrm{l}\:\mathrm{c}\mathrm{o}\mathrm{m}\mathrm{m}\mathrm{u}\mathrm{n}\mathrm{i}\mathrm{t}\mathrm{y}}\times\:100$$

#### Lodging Index (LI)

Six uniformly vigorous plants were selected from each plot. The breaking strength of the second basal internode (BS₂) was measured using a YYD-1 stem strength tester (Zhejiang Tuopu Technology Co., Ltd., Hangzhou, China). After removing the leaf sheaths, the bare internode was placed on the instrument’s supports (set to a 2 cm span). The probe applied vertical pressure at a constant rate until fracture occurred, and the peak force recorded was defined as BS₂. The LI was then calculated using HCG, AFW, and BS₂ [42]:

$$\:LI=\frac{HCG\times\:\mathrm{A}\mathrm{F}\mathrm{W}}{{BS}_{2}}$$

#### Determination of stem physicochemical traits

Upon harvest at the milk stage, the second basal internodes were immediately subjected to thermal deactivation (oven-dried at 105 °C for 30 min) and subsequently dried at 65 °C to a constant weight. The dried samples were ground into a fine powder (passing through a < 60 mesh sieve). This prepared material was used to quantify starch (ST), soluble sugars (SS) [43], soluble protein (SP) [44], calcium (Ca), potassium (K), magnesium (Mg), silicon (Si) [45, 46], cellulose content (CC) [47], lignin content (LC) [48].

### Statistical analysis

Data processing and integration were performed using Microsoft Excel 2019 and R software (version 4.0.2). Differences between treatment means were evaluated via one-way Analysis of Variance (ANOVA) followed by Duncan’s multiple range test (*P* < 0.05). To elucidate the regulatory pathways through which variety, N dosage, and their interactions influence the oat lodging index, a piecewise structural equation model (SEM) was constructed to calculate the respective path coefficients. Furthermore, the TOPSIS algorithm was employed to comprehensively evaluate stem physicochemical traits alongside lodging risks, facilitating the identification of the optimal N strategy for this region. All graphical representations were generated using R software and OriginPro 2024.

## Data Availability

The data and images that support the findings of this study are available from the corresponding author upon reasonable request.
